# Loss of bacterial diversity in the sinuses is associated with lower smell discrimination scores

**DOI:** 10.1038/s41598-020-73396-3

**Published:** 2020-10-02

**Authors:** Kristi Biswas, Brett Wagner Mackenzie, Charlotte Ballauf, Julia Draf, Richard G. Douglas, Thomas Hummel

**Affiliations:** 1 Department of Surgery, University of Auckland, 85 Park Road, Grafton, Auckland, 1023 New Zealand; 2 Smell and Taste Clinic, ENT Department, Technische Universität Dresden, Dresden, Germany

**Keywords:** Microbiology, Microbiome, Respiratory signs and symptoms

## Abstract

Olfactory impairment affects ~ 20% of the population and has been linked to various serious disorders. Microbes in the nasal cavity play a key role in priming the physiology of the olfactory epithelium and maintaining a normal sense of smell by the host. The aim of this study was to explore the link between olfactory dysfunction and nasal bacterial communities. A total of 162 subjects were recruited for this study from a specialized olfactory dysfunction clinic and placed into one of three groups: anosmia, hyposmia or normosmia. Swabs from the nasal middle meatus were collected from each subject then processed for bacterial 16S rRNA gene sequencing. No overall differences in bacterial diversity or composition were observed between the three cohorts in this study. However, the relative abundances of *Corynebacterium* spp. and *Streptococcus* spp. were significantly (*p* < 0.05) different in subjects with olfactory loss. Furthermore, subjects with deficiencies in discriminating between smells (based on discrimination scores) had a lower bacterial diversity (Simpson’s evenness *p* < 0.05). While these results are preliminary in nature, potential bacterial biomarkers for olfactory loss were identified. These findings need to be further validated and biologically tested in animal models.

## Introduction

The sense of smell is an important part of daily life. It helps guide behaviour, eating habits, detection of danger and taste^1^. Olfactory dysfunction generally increases with age, but can also be caused by head trauma, sinonasal disease, infection to the upper respiratory tract (URT) or neurodegenerative disorders^2^. One recent study reported that a loss of smell can be an early predictor for 5-year mortality in individuals without dementia aged 40–90^3^. Changes in body weight have also been shown to be linked to olfactory dysfunction, but the exact effect is still not clear^4,5^.

A complete loss of smell (anosmia) or partial loss (hyposmia) affects approximately 20% of the global population^6^. After an URT infection about 2/3 of the affected population spontaneously recovers, while those with chronic inflammatory diseases like chronic rhinosinusitis can often be treated successfully with corticosteroids or sinus surgery. During periods of chronic inflammation, olfactory stem cells forgo their normal function and help with pathogen removal and immune defense leading to loss of smell^7^. While recovery of smell can be a slow process, ‘smell training’ was introduced in the last 10 years for certain types of olfactory disorders to expedite the process^8,9^.

Although recent studies suggest that the microbiota in the sinonasal cavity of mice can modulate the physiology of olfactory epithelium^10^, few studies linking the sinonasal microbiota and olfaction in humans have been published. One recent study compared the sinonasal bacterial composition between healthy subjects with hyposmia (n = 10), normosmia (n = 28), and slightly higher olfactory function (n = 29). The results showed that individuals with slight olfactory impairment had elevated relative abundances of the bacterial families *Comamonadaceae* and *Enterobacteriaceae*, while the genera *Corynebacterium* and *Faecalibacterium* were reduced^11^. However, this study could have benefited with the inclusion of an anosmia cohort.

Evidence for the potential link between oral and nasal microorganisms, the olfactory bulb (as an entry zone from the nasal cavity to the brain) and neurological diseases is increasing^12,13^. These studies, along with others demonstrating a microbial dysbiosis in the sinuses of respiratory diseases^14,15^, signify that the microbial community has the potential to reflect the sensory function and health status of an individual.

Identifying potential early predictors of respiratory diseases has gained momentum because it allows for earlier treatment intervention which can result in less severe disease^16^. The identification of early microbial predictors for olfactory dysfunction may help with earlier medical interventions and better patient outcomes^17^. In this study, we aim to investigate the association of the sinus bacterial community composition with loss of smell using molecular methods.

## Results

In the final dataset, samples from 120 patients across 418 amplicon sequence variants (ASV)s were analysed. During the rarefaction process, the DNA extraction control samples and PCR negative samples were filtered out of the dataset due to low read counts or poor quality sequences. Unless otherwise stated, the quality filtered, rarefied ASV table was used for all downstream processing.

Subjects were divided into 3 cohorts based on their threshold, discrimination and identification (TDI) scores: anosmia (n = 32), hyposmia (n = 57), and normal (n = 31). There was significantly greater number of hyposmia subjects in this study compared to the other two cohorts (chi-square *p* = 0.004). Of all the clinical parameters and patient data collected, age, etiology, and duration of disease were significantly different between the cohorts (Table 1). The cohort with a normal sense of smell was significantly younger than the other two cohorts. If patients had any underlying conditions such as acute sinusitis or tonsillitis symptoms this was recorded; subjects in the hyposmia cohort had a significantly greater incidence of these underlying conditions. Accordingly, adjustments of false-discovery rates were made during statistical analysis of relative abundance of microbial ASVs between the three cohorts.

**Table 1:** Patient demographics and results from statistical analyses.

Variables	Normal (n = 31)	Hyposmia (n = 57)	Anosmia (n = 32)	Unadjusted test *p*-value
Female	16/31	34/57	17/32	NS
Never smoked	28/31	54/57	31/32	NS
Age at sampling	48.6 ± 17.1	61.3 ± 11.7	65.0 ± 11.2	***p < 0.05***
Parkinson’s in the family (Yes)	4/31	1/57	3/32	NS
Alzheimer’s in the family (Yes)	4/31	4/57	3/32	NS
**Etiology**				
Healthy control	24	0	0	***p < 0.05***
Idiopathic	1	21	17	
Post viral	6	36	15	
**Duration of symptoms (months)**				
0 -6	28	28	18	***p < 0.05***
> 6	3	29	14	
Hypertension (yes)	8	23	15	NS
Tonsil disease (yes)	3	10	4	NS
Sinus disease (yes)	2	3	3	NS
Disease prevalence (yes)	12	40	25	***p < 0.01***

Continuous variables were tested for normality using Shapiro–Wilk normality test followed by analysis of variance then Tukey multiple comparisons of means for pairwise comparisons. Means ± standard deviation are shown. Categorical variables were tested using a chi square test. *p* < 0.05 is considered statistically significant and significant results are shown in bold typeface. Patients are categorised based on TDI scores; whereby a total TDI score > 30.5 indicates normal sense of smell, 16.5–30.5 indicates hyposmia, and < 16.5 indicates anosmia. NS = not significant, *p* > 0.05.

### Overall bacterial community analyses

**Figure 1:**
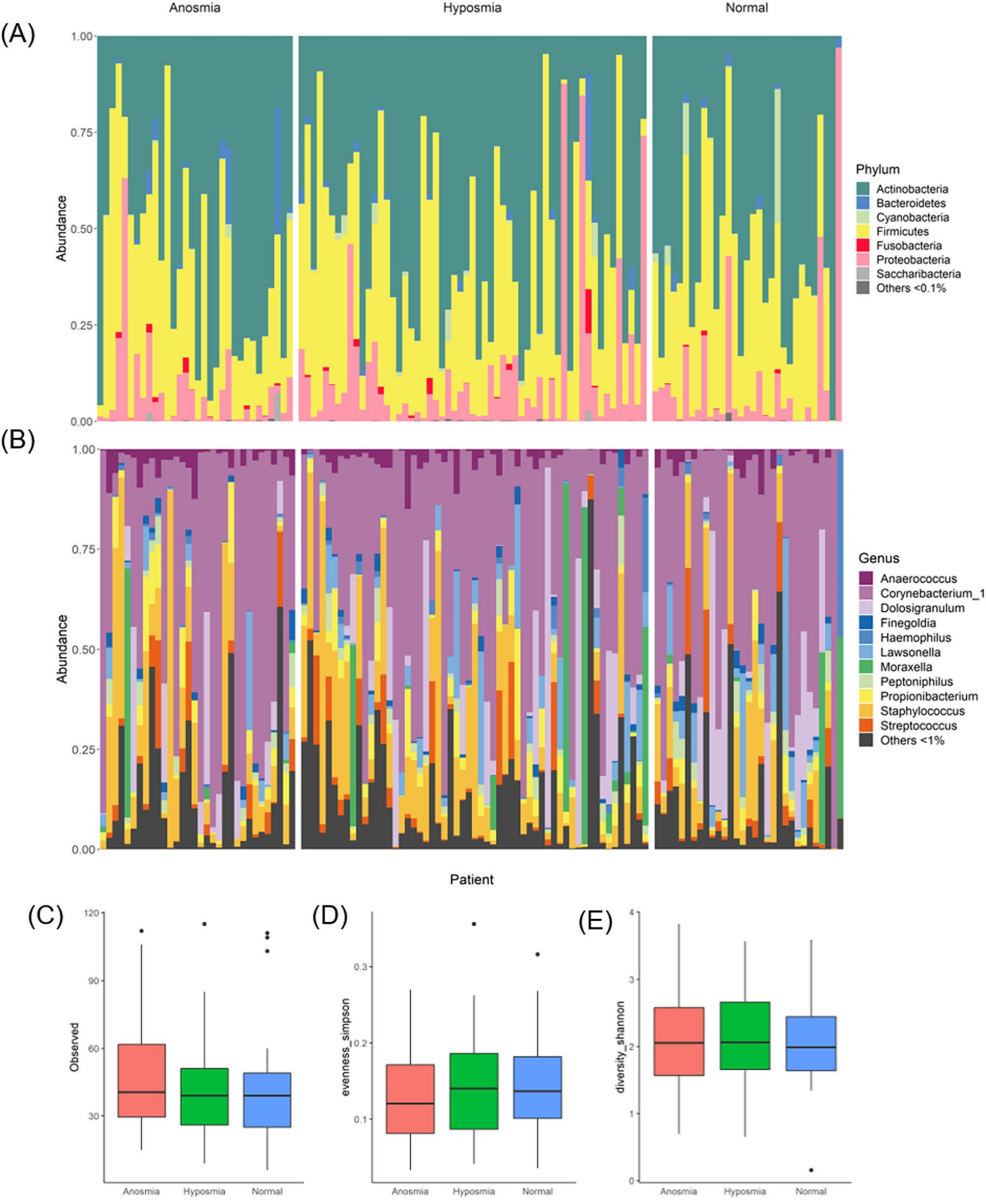
Bacterial community data at the (**A**) phylum and (**B**) genus levels for each subject. Subjects are grouped based on TDI scores (anosmia, hyposmia and normal). Box and whisker plots to visualise the relative abundance changes were generated using ‘ggplot2’^36^, and represent grouped summaries for alpha diversity metrics: (**C**) Observed amplicon sequence variants, (**D**) Simpson’s evenness, and (**E**) Shannon diversity.

Across all subjects, the bacterial communities were dominated by phyla *Actinobacteria* and *Firmicutes*. At the genus level, the majority of the sequences belonged to *Corynebacterium* and to lesser extent *Staphylococcus*, *Dolosigranulum*, and *Moraxella* (Fig. 1A,B). The effects of all measured clinical factors on the bacterial communities were tested through ‘adonis’ analysis. Only gender (R^2^ = 1.7%) and smoking status (R^2^ = 2.3%) significantly (*p* < 0.05) contributed to the variation observed in the bacterial community composition.

There were no significant differences in any of the alpha diversity measures between TDI groups (Fig. 1)C–E), or when subjects were grouped based on BMI, etiology, gender, smoking status, and duration of symptoms.

### Specific bacterial ASV analyses

Pairwise comparisons of individual ASVs revealed that the relative abundances of *Streptococcus*, *Blastomonas*, *Anaerococcus*, *Lawsonella*, *Nocardioides*, *Corynebacterium*, *Fusobacterium*, and *Staphylococcus* were significantly different between the TDI grouped cohorts (Table 2). Interestingly, *Streptococcus* ASV111 and *Anaerococcus* ASV43 were significantly more abundant in the anosmia group compared to the other two cohorts. Whereas *Streptococcus* ASV65 and *Corynebacterium* ASV46 were significantly more abundant in the normal cohort compared to the hyposmia group. The relative abundances of other significantly different ASVs listed in Table 2 were substantially lower (< 0.2% of the overall sequence abundance).

**Table 2:** Dunn’s test pairwise comparisons of individual amplicon sequence variants (ASVs) (overall abundance > 0.01%) between SDI categories.

ASV	Phylum	Genus	Anosmia–Hyposmia	Anosmia–Normal	Hyposmia–Normal
ASV111	*Firmicutes*	*Streptococcus*	**0.009**	**0.001**	**–**
ASV456	*Proteobacteria*	*Blastomonas*	**0.005**	**0.005**	**–**
ASV65	*Firmicutes*	*Streptococcus*	**–**	**–**	**0.007**
ASV200	*Actinobacteria*	*Lawsonella*	**–**	**0.013**	**0.013**
ASV46	*Actinobacteria*	*Corynebacterium_1*	**–**	**–**	**0.008**
ASV544	*Fusobacteria*	*Fusobacterium*	**0.012**	**0.020**	**–**
ASV360	*Firmicutes*	*Anaerococcus*	**0.013**	**0.030**	**–**
ASV827	*Actinobacteria*	*Nocardioides*	**–**	**–**	**0.013**
ASV43	*Firmicutes*	*Anaerococcus*	**0.018**	**–**	**–**
ASV160	*Firmicutes*	*Staphylococcus*	**–**	**–**	**0.025**
ASV202	*Firmicutes*	*Staphylococcus*	**–**	**0.023**	**–**

Only values that were significant (*p* < 0.05).

### Individual TDI category analyses

**Figure 2:**
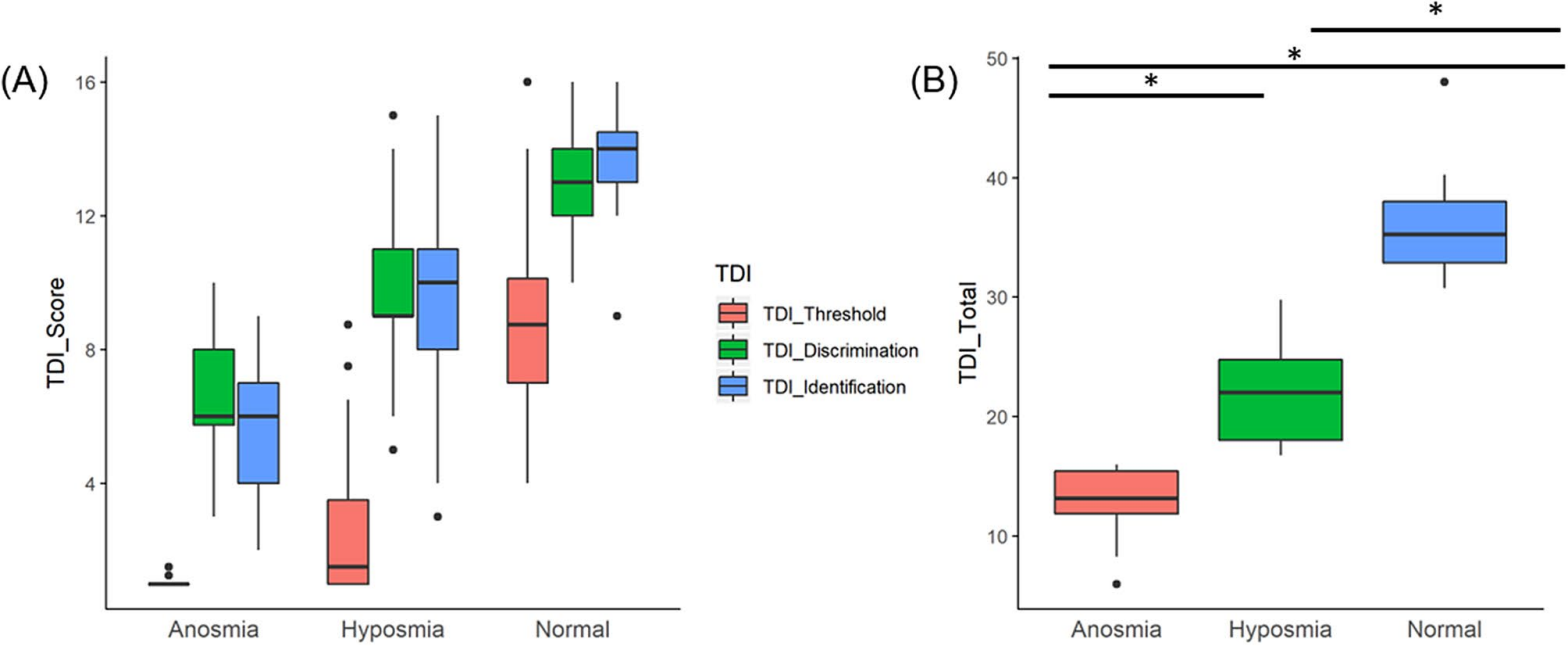
Results of the smell test (TDI) for the subjects of this study are shown in this graph. Box and whisker plots used visualise the relative abundance changes were generated using ‘ggplot2’^36^ show threshold, discrimination and identification scores for each cohort separately (**A**) and as a combined total TDI score (**B**). Significant differences (*p* < 0.05 indicated with *) were observed between each cohort.

Scores used to categorise patients as anosmic, hyposmic or normal were based on independent threshold, discrimination and identification (TDI) criteria. We observed significant differences in the three independent TDI scores between the cohorts (anosmia, hyposmia and normal) (Fig. 2).

**Figure 3:**
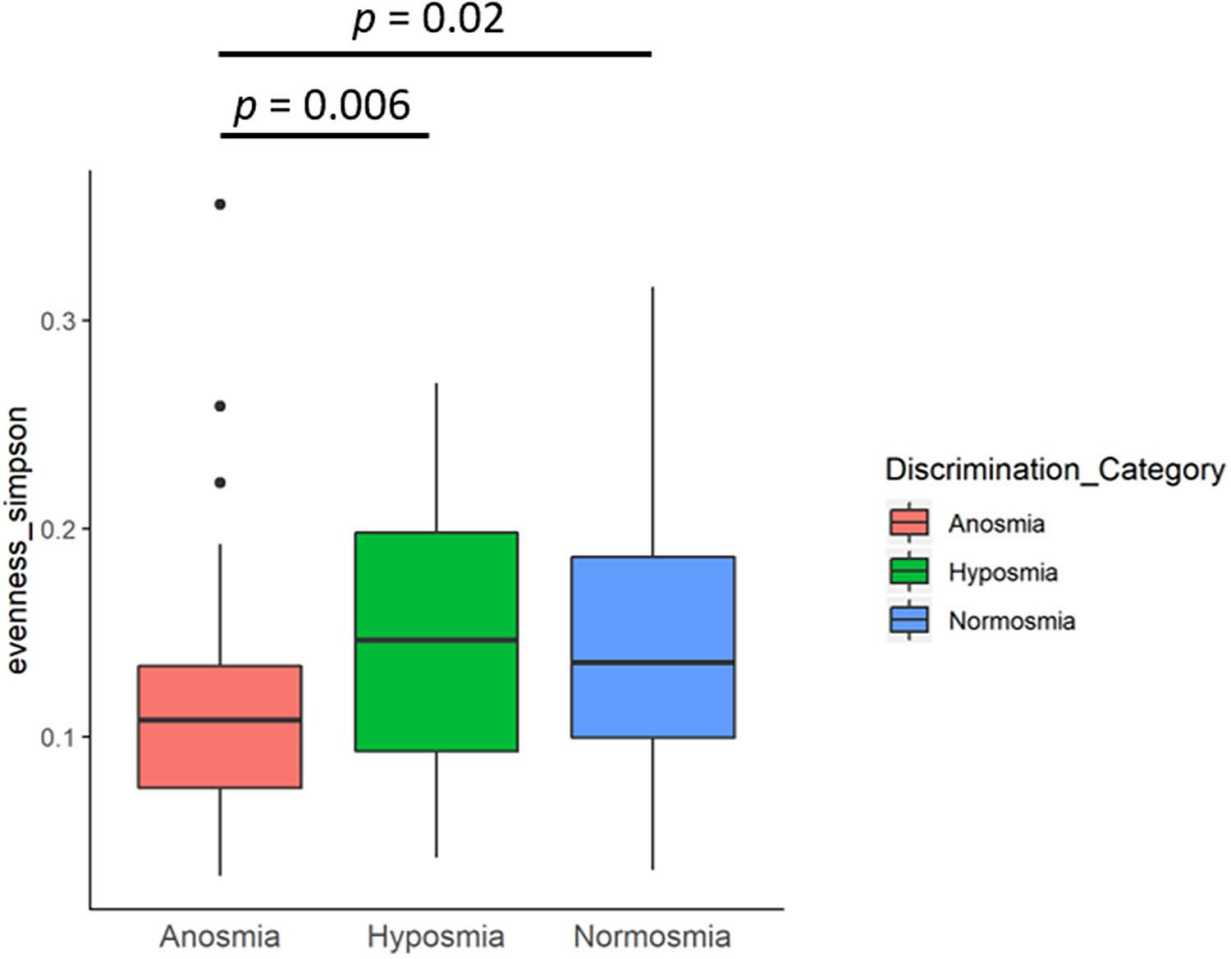
Simpson’s evenness alpha diversity results for the three cohorts of this study (anosmia, hyposmia and normal) based on discrimination scores only are shown. Grouped summaries for each cohort are represented in the box and whisker plots generated using ‘ggplot2’^36^. Significant differences were observed between anosmia subjects and the other two cohorts.

Furthermore, when we analysed the bacterial communities based on the independent TDI scores, we observed a significant difference in the Simpson’s evenness alpha diversity metric. This difference was only observed when subjects were categorized based on discrimination scores (Fig. 3). Specifically, the anosmia cohort had significantly reduced diversity when compared to the other two cohorts. The pairwise analysis of individual ASVs based on the discrimination scores are shown in Table 3. Comparisons of taxa between groups based on discrimination scores revealed that hyposmic and normosmic groups had the highest number of ASVs that were significantly different in terms of relative abundances. *Streptococcus* ASV111 was again found to be significantly greater in the anosmia group and *Corynebacterium* ASV265 was found to be significantly greater in the normal cohort.

**Table 3:** Dunn’s test pairwise comparisons of individual amplicon sequence variants (ASVs) (overall abundance > 0.01%) between discrimination scores only.

ASV	Phylum	Genus	Anosmia–Hyposmia	Anosmia–Normosmia	Hyposmia–Normosmia
ASV360	*Firmicutes*	*Anaerococcus*	**0.000**	**0.003**	**–**
ASV111	*Firmicutes*	*Strepotococcus*	**0.039**	**0.001**	**0.037**
ASV36	*Proteobacteria*	*Campylobacter*	**0.019**	**–**	**0.006**
ASV200	*Actinobacteria*	*Lawsonella*	**–**	**0.012**	**0.005**
ASV78	*Firmicutes*	*Veillonella*	**0.029**	**–**	**0.010**
ASV203	*Proteobacteria*	*Brevundimonas*	**0.008**	**0.013**	**–**
ASV137	*Proteobacteria*	*Tepidimonas*	**–**	**–**	**0.005**
ASV126	*Firmicutes*	*Lactobacillus*	**0.015**	**–**	**0.018**
ASV544	*Fusobacteria*	*Fusobacterium*	**0.011**	**0.012**	**–**
ASV265	*Actinobacteria*	*Corynebacterium_1*	**–**	**0.018**	**0.012**
ASV487	*Proteobacteria*	*Haemophilus*	**0.014**	**0.016**	**–**
ASV87	*Bacteroidetes*	*Prevotella_7*	**–**	**–**	**0.019**
ASV922	*Firmicutes*	*Streptococcus*	**0.026**	**–**	**0.039**
ASV149	*Firmicutes*	*Gemella*	**–**	**–**	**0.023**
ASV52	*Firmicutes*	*Streptococcus*	**–**	**0.038**	**0.022**
ASV202	*Firmicutes*	*Staphylococcus*	–	**0.023**	**0.035**

Only values that were significant (*p* < 0.05) are shown.

Despite the lack of significant differences in overall alpha diversity across the three cohorts for independent threshold or identification scores, each category had a different set of microbial variants that differed significantly (Supplementary Tables 1 and 2).

## Discussion

The human microbiome is an integral part of maintaining homeostasis in a healthy individual. Microorganisms associated with the human body can also play an important role in many diseases and disorders across various body sites. There is a spectrum of microbe-host related interactions; these range from a single pathogen infection such as *S. aureus* causing skin infections to overall community structure imbalances in the gut like those observed in obese individuals^18,19^. The link between olfactory loss and the sinonasal microbiome has been little studied. In this cross-sectional observational study we investigated the overall bacterial community composition and diversity, along with shifts in sequence abundances of individual bacterial taxa across patients with normal sense of smell to complete loss of smell. This study adds new knowledge to the current literature as it is the largest study to date examining the bacterial composition in olfactory loss, and importantly, we included an anosmia group which was lacking in earlier studies.

A loss of smell is a natural process in the older population (> 60 years)^20^ and this is evident in our study cohort. However, this confounding factor was accounted for when comparing the microbial communities between the three groups of this study. The sinonasal bacterial profiles detected from the samples in this study are consistent with those previously detected in the sinuses using 16S rRNA amplicon sequencing^14,21,22^. Specifically, a dominance of members from the genera *Corynebacterium*, *Staphylococcus*, *Moraxella* and *Dolosigranulum* were observed. Our study found no significant differences in the overall bacterial community composition and diversity between anosmic, hyposmic and normal subjects. In contrast to our findings, a recent study reported an increase in bacterial diversity and function in subjects with hyposmia compared to subjects with a normal sense of smell^11^. However, all participants recruited for that study were healthy, and only a small proportion had a slight decrease in olfactory function when tested. In our present study, hyposmic and anosmic patients experienced olfactory loss for a sustained period of time and sought specific clinical counseling for their condition.

While no overall differences in bacterial diversity or richness were observed between the three cohorts, the relative sequence abundances of two particular ASVs (associated with *Streptococcus* and *Corynebacterium genera)* were associated with loss of smell. A recent study comparing the nasal microbiome of Parkinson’s disease patients with olfactory loss reported similar results, with no overall differences in bacterial diversity^12^. Additionally, the relative abundances of taxa such as *Moraxella* and *Staphylococcus* were significantly associated with a loss of olfactory function. These subtle differences in the bacterial community could initiate or exacerbate changes in olfactory function. Further research to test the biological mechanisms of these two identified microbes of interest and olfactory dysfunction will need to be carried out.

Sub-setting the individual components of smell categories which combine multiple facets offers increased insight into the nuances associated within those categories. In our study, we divided the individual TDI scores (threshold, discrimination and identification), and then independently analysed the bacterial data in relation to these scores. Threshold was previously identified as the category which contributes the most to the differences in bacterial communities between TDI scores^11^. However, in this study we found that the discrimination score was the only TDI category to have a significant impact on diversity measures. Subjects with a complete or partial loss of smell had significantly lower bacterial diversity than subjects with a normal sense of smell. This finding is consistent with other disorders and diseases in human microbiome research, but is in contrast to the previous study which reported increased diversity in subjects with partial smell impediments compared to normosmic participants. Interestingly, the abundances of *Corynebacterium* spp. were significantly reduced in subjects with olfactory loss in both our study and previous studies^11^. We propose that some species from the *Corynebacterium* genus may be potential biomarkers for loss of olfactory function. Further studies which quantify and identify *Corynebacterium* species or strains should explore this possibility. Ultimately, identifying microbial biomarkers for olfactory loss could help identify those patients who may be at risk for losing their sense of smell and facilitate an earlier intervention. Earlier treatments often result in better prognosis for patients^23,24^.

In light of the results from this study, different bacterial biomarkers may exist for each of the different smell categories. Furthermore, we speculate that due to this reason a global difference in overall TDI score was not observed. The lack of any significant correlation of bacterial communities for the three cohorts and measures such as BMI, etiology, and history of other illness was surprising as these correlations have been reported previously^11^. We speculate that if samples were collected from the olfactory cleft instead of the middle meatus then differences in the microbial communities would become more apparent. Sampling from the sinuses is one of the limitations of this study. We recommend that a study examining variation in bacterial community composition between sinonasal and olfactory cleft sample sites should be conducted. Many such studies have been done previously (including one from our own group), but these studies did not include olfactory cleft samples^25,26^.

## Conclusions

In this study, we explored the link between olfactory function and sinonasal bacterial community composition. Although few differences in bacterial community composition between the three cohorts in this study were found, important observations were made through our analyses. We found that the relative abundances of amplicon variants associated with *Streptococcus* and *Anaerococcus* genera were significantly more abundant in the anosmia group compared to the other two cohorts. Analyses focusing on changes in the relative abundances of specific ASVs revealed different smell categories have different bacterial biomarkers. Additionally, when we examined cohorts based on the individual components of the TDI scoring system, we found that the loss of discriminating smells was correlated with decreased bacterial diversity in the sinuses. Future work should focus on establishing a biological link between olfactory dysfunction and species of *Streptococcus* and *Corynebacterium*.

## Methods

### Subject clinical information

**Figure 4:**
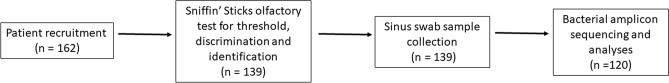
A schematic of the study design.

In total, 162 individuals were recruited that visited the specialized olfactory dysfunction clinic in Dresden, Germany. A schematic of the study design is shown in Fig. 4. Patients were categorised based on TDI scores: a total TDI score ≥ 30.5 indicated a normal sense of smell, 16.5–30.5 indicated hyposmia, and < 16.5 indicated functional anosmia (further termed “anosmia”). Subjects’ body mass index (BMI), age at sampling, gender, smoking status, duration of disease, etiology, current medication, family history of neurodegenerative disorders, head trauma, and other co-morbidities were recorded based on a standardized, structured medical history^27^. Subjects that were < 18 years of age, pregnant, or had taken antibiotics in the 4 weeks prior to sample collection were excluded from the study.

This study had approval from the ethics committee at the Medical Faculty of the TU Dresden (ethics # EK21012018). Written informed consent was collected from all participating subjects. All experiments were performed in accordance with relevant guidelines and regulations.

### TDI test details

In an established clinical test of olfactory acuity different odors were administered with pen-like odor dispensers ("Sniffin' Sticks")^28^. For olfactory presentation, the pen’s cap is removed for about 3 s, its tip is placed in front of the subject's nose and carefully moved from the left to the right nostril and back. The olfactory threshold value for phenylethyl alcohol (PEA; dissolved in propylene glycol) was determined in a three-alternative forced choice (3-AFC) paradigm, during which participants repeatedly received triplets of pens. They had to distinguish the pen containing a certain concentration of PEA from two blanks filled with the solvent. The highest concentration was a 4% odor solution. Sixteen concentrations (dilution ratio 1:2) were presented starting from the lowest odor concentration using a staircase paradigm. Two consecutive correct identifications of the odor or one incorrect answer marked a so-called turning point and led to a decrease or increase in odor concentration. The triplets were presented at intervals of 20 s. The threshold value was the mean value of the last four turning points in the staircase, with the final score ranging between 1 and 16 points. The same 3-AFC logic was used for the odor discrimination task. Two pens of any triplet contained the same odor while the third pen smelled different. The subjects were asked to indicate the pen with a different smell. The odor presentation interval within a triplet was about 3 s. The intervals between the triplets were 20 s. The score was the sum of the correctly identified odors. Therefore, the score in this task ranged from 0 to 16 points. Subjects were blindfolded during the threshold and discrimination tasks to avoid visual identification of the target pens. Odor identification included common and familiar smells (recognized by at least 75% of the population). Using a 4-AFC task the subjects were asked to identify the smells from lists of four verbal descriptors. The interval between presentation of pens was about 20 s. The total score was the sum of the correctly identified pens, so that the test subjects scored between 0 and 16 points. The final "TDI score" was the sum of the scores for the subtests "threshold", "discrimination" and "identification".

### Sample collection and DNA extraction

During clinic, pairs of sterile rayon-tipped swabs (Copan, #170KS01) were collected from the left middle meatus of each subject by a trained clinician. If the clinician suspected a possibility of contamination by touching other nasal sites then swabs were discarded and samples recollected from the middle meatus. Using sterile techniques, the tip of each swab was placed in a sterile 2 mL screw-capped tube containing RNALater® nucleic acids preservative. Tubes were stored at room temperature for 24 h before being transferred to − 20 °C for storage. Once all samples had been collected for this study, they were sent to the laboratory at the University of Auckland, New Zealand for further analyses.

As previously described^14,25^ pairs of swabs from each subject were thawed on ice and placed together into a sterile Lysing Matrix E tube (MP Biomedicals, Australia). Cells were ruptured using a Bead Beater at 2.9 m/s for 2 × 30 s. Genomic DNA was extracted from the samples using the AllPrep DNA/RNA Mini Kit (Qiagen) following the manufacturer's instructions and eluted in 30 μL of DNase-free water. The quality and quantity of genomic DNA were measured on a Nanodrop 3300 fluorospectrometer. A negative DNA extraction control containing 200 µL of sterile PCR-grade water was carried out simultaneously to assess the kit for contamination.

### PCR amplification and sequencing

The bacterial communities for each sample was processed as described previously^22^. In brief, the V3-V4 region of the bacterial 16S rRNA gene was amplified using the S-D-Bact-0341-b-S-17/S-D-Bact-0785-a-A-21^29^ primer pair containing Nextera library prep kit adapters. Approximately 100 ng of genomic template DNA was used in duplicate PCRs, each consisting of 35 cycles. Negative PCR controls were included in all PCR reactions as well as elute from the negative extraction control, which yielded no detectable products. Duplicate PCR products were pooled to a final volume of 50 µL and purified using Agencourt AMPure magnetic beads (Beckman Coulter Inc., USA) according to the manufacturer’s instructions. Purified PCR products were quantitatively assessed with Qubit dsDNA high-sensitivity kits (Life Technologies, New Zealand) and standardised to ~ 5 ng per sample. The purified products were submitted to the Auckland Genomics Centre for library preparation and sequencing on the Illumina MiSeq 2 × 300 base pair platform with pair end reads. Raw sequence reads are stored on a publicly available database (NCBI) under BioProject number PRJNA638970.

### Bacterial 16S rRNA gene sequence data processing

Of the original 162 samples processed for sequencing, data were analysed for 139 patients after reviewing symptoms and etiology. Specifically, those patients whose diagnosis and TDI scores did not match or had a history of CRS were removed as these data could confound conclusions. Data were processed according to version 1.12 DADA2 pipeline in R^30^. Most parameters were kept as default, except ‘truncLen’ was set to 280 and 240 for forward and reverse reads, respectively, and primers were removed. Quality filtered sequences that were < 300 bp or > 430 bp were considered non-target and removed from the dataset, then chimeras were identified and removed. Taxonomy was assigned to amplicon sequence variants (ASVs) using the SILVA nonredundant v128 database^31^. Non-target Eukaryote taxa were removed prior to the removal of ASVs with a prevalence less than 3 times in at least 5% of the samples. The resulting data were then rarefied to 1600 counts per sample. Rarefaction excluded sinus samples from 19 patients. The final dataset for downstream processing included 418 taxon-assigned ASVs across 120 samples.

### Data analysis and statistics

All data analyses and statistics were carried out in R version 3.6.0^32^. Continuous variables for the patient data were tested for normality using Shapiro–Wilk normality test followed by analysis of variance then Tukey multiple comparisons of means for pairwise comparisons. The bacterial communities and assessment scores for each independent category (threshold, discrimination, identification) that comprises the total TDI scores were evaluated independently with pairwise comparisons for each of the three cohorts (anosmia, hyposmia and normal). Categorical variables for the patient data were tested using a chi- square test. Values of *p* < 0.05 were considered statistically significant and significant results are shown in bold typeface in Table 1.

Alpha diversity (diversity within samples) metrics were calculated in the R package ‘microbiome’^33^. Beta-diversity (diversity between samples) was calculated in R using the ‘vegan’ package^34^. The Bray–Curtis dissimilarity index was chosen for its ability to detect underlying ecological gradients^35^. Permutational multivariate analyses of variance based on Bray–Curtis distance matrices were conducted using the ‘adonis’ command in the ‘vegan’ package. Finally, statistical tests were conducted to evaluate ASVs which exhibited a significant change in relative abundance between groups and subsets of data after the adjustment for false discovery rate. ASVs with < 0.01% total relative abundance were removed before comparisons. For categorical variables with > 3 groups, Dunn’s test was conducted with Benjamini–Hochberg multiple pairwise corrections in the R package ‘dunn.test’ to provide adjusted *p* values. The student’s *t*-test was applied for categorical variables with two groups. Box plots to visualise the relative abundance changes in Figs. 1, 2 and 3 were generated using ‘ggplot2’^36^.

## Supplementary information

Supplementary Tables.
