# Supplementary material for: Loss of bacterial diversity in the sinuses is associated with lower smell discrimination scores
Source: Sci Rep. 2020 Oct 2;10:16422. doi: 10.1038/s41598-020-73396-3 (PMC7532173; doi:10.1038/s41598-020-73396-3)
Supplement: Supplementary file 1 — Supplementary Tables. [file 41598_2020_73396_MOESM1_ESM.pdf]

Loss of bacterial diversity in the sinuses is associated with lower smell discrimination scores

Kristi Biswas[1*], Brett Wagner Mackenzie[1], Charlotte Ballauf[2], Julia Draf[2], Richard G. Douglas[1], Thomas Hummel[2]

[1]Department of Surgery, University of Auckland, New Zealand

[2]Smell and Taste Clinic, ENT Department, Technische Universität Dresden, Germany

Supplementary Table 1: Dunn's test pairwise comparisons of individual ASVs (overall abundance >0.01%) between threshold scores only.
Only values that were significant ($p < 0.05$) are shown below.

ASV	Phylum	Genus	Anosmia - Hyposmia	Anosmia - Normosmia	Hyposmia - Normosmia
ASV111	*Firmicutes*	*Streptococcus*	**0.012**	**0.001**	**-**
ASV46	*Actinobacteria*	*Corynebacterium_1*	**-**	**0.030**	**0.001**
ASV193	*Proteobacteria*	*Paracoccus*	**0.020**	**-**	**0.006**
ASV128	*Firmicutes*	*Anaerococcus*	**0.006**	**0.034**	**-**
ASV544	*Fusobacteria*	*Fusobacterium*	**0.017**	**0.014**	**-**
ASV202	*Firmicutes*	*Staphylococcus*	**-**	**0.008**	**-**
ASV2	*Firmicutes*	*Dolosigranulum*	**-**	**0.018**	**0.011**
ASV462	*Proteobacteria*	*Paracoccus*	**0.030**	**-**	**0.013**
ASV340	*Firmicutes*	*Anaerococcus*	**0.013**	**0.046**	**-**
ASV387	*Proteobacteria*	*Paracoccus*	**0.014**	**0.047**	**-**
ASV1	*Actinobacteria*	*Corynebacterium_1*	**-**	**0.021**	**0.015**
ASV360	*Firmicutes*	*Anaerococcus*	**0.014**	**-**	**-**
ASV269	*Actinobacteria*	*Kocuria*	**0.044**	**0.018**	**-**
ASV109	*Proteobacteria*	*Unassigned (Neisseriaceae)*	**0.015**	**-**	**-**
ASV198	*Firmicutes*	*Staphylococcus*	**0.015**	**-**	**-**
ASV442	*Proteobacteria*	*Unassigned*	**0.025**	**0.036**	**-**
ASV302	*Proteobacteria*	*Vulcaniibacterium*	**-**	**-**	**0.021**

Supplementary Table 2: Dunn's test pairwise comparisons of individual ASVs (overall abundance >0.01%) between identification scores only.
Only values that were significant ($p < 0.05$) are shown below.

ASV	Phylum	Genus	Anosmia - Hyposmia	Anosmia - Normosmia	Hyposmia - Normosmia
ASV149	*Firmicutes*	*Gemella*	-	**0.003**	-
ASV97	*Firmicutes*	*Streptococcus*	-	**0.015**	**0.015**
ASV277	*Firmicutes*	*Stomatobaculum*	**0.024**	-	**0.010**
ASV279	*Firmicutes*	*Staphylococcus*	**0.028**	**0.012**	-
ASV273	*Firmicutes*	*Broncothrix*	**0.016**	-	**0.019**
ASV413	*Actinobacteria*	*Unassigned Intrasporangiaceae*	-	**0.031**	**0.016**
ASV65	*Firmicutes*	*Streptococcus*	-	**0.018**	**0.047**
ASV52	*Firmicutes*	*Streptococcus*	-	-	**0.014**
ASV36	*Proteobacteria*	*Campylobacter*	-	-	**0.017**
ASV96	*Bacteroidetes*	*Alloprevotella*	**0.022**	**0.031**	-
ASV345	*Firmicutes*	*Oribacterium*	**0.020**	-	**0.036**
ASV118	*Firmicutes*	*Veillonella*	-	**0.022**	-
ASV122	*Firmicutes*	*Veillonella*	-	**0.022**	**0.044**
ASV383	*Proteobacteria*	*Rhizobium*	-	-	**0.018**
ASV69	*Firmicutes*	*Staphylococcus*	-	**0.020**	-
ASV116	*Proteobacteria*	*Escherichia/Shigella*	-	-	**0.021**
ASV102	*Actinobacteria*	*Brevibacterium*	**0.031**	-	**0.046**
